# From pictures to reality: modelling the phenomenology and psychophysics of 3D perception

**DOI:** 10.1098/rstb.2021.0454

**Published:** 2023-01-30

**Authors:** Dhanraj Vishwanath

**Affiliations:** School of Psychology and Neuroscience, University of St Andrews, St Andrews, Fife KY16 9JP, UK

**Keywords:** 3D perception, depth perception, stereopsis, picture perception, phenomenology, affordance and embodiment

## Abstract

The dominant inferential approach to human 3D perception assumes a model of spatial encoding based on a physical description of objects and space. Prevailing models based on this physicalist approach assume that the visual system infers an objective, unitary and mostly veridical representation of the external world. However, careful consideration of the phenomenology of 3D perception challenges these assumptions. I review important aspects of phenomenology, psychophysics and neurophysiology which suggest that human visual perception of 3D  objects and space is underwritten by distinct and dissociated spatial encodings that are optimized for specific regions of space. Specifically, I argue that 3D perception is underwritten by at least three distinct encodings for (1) egocentric distance perception at the ambulatory scale, (2) exocentric distance (scaled depth) perception optimized for near space, and (3) perception of object shape and layout (unscaled depth). This tripartite division can more satisfactorily account for the phenomenology, psychophysics and adaptive logic of human 3D perception.

This article is part of a discussion meeting issue ‘New approaches to 3D vision’.

## 1. Introduction

Textbook descriptions of the psychology of 3D visual perception most often claim that the visual system acts as a sort of ‘ideal observer’ that faithfully infers and then ‘re-presents’ the physical 3D geometric structure of objects and space that is objectively given in the external world. This approach typically eschews the analysis of the phenomenology of perception. Moreover, it views systematic errors in spatial perception, not as indicative of peculiarities in the encoding of spatial parameters themselves, but instead, simply the result of noisy or impoverished sensory signals. In this opinion article I will outline how both phenomenological and psychophysical evidence is largely inconsistent with this conventional model and, instead, argues for a model of 3D space perception consisting of multiple, sometimes mutually inconsistent, encodings of space. Specifically, I will argue for the existence of three major distinct spatial encodings that can account for both empirical results and phenomenological observations. In this introductory section, I will discuss the broad conceptual difference between the prevailing inferential/representational approach and the alternative phenomenological approach to 3D perception. In §2, I will discuss in more detail the phenomenology and psychophysics of 3D space perception to argue why the inferential/representational approach fails. In §3, I will discuss the alternative phenomenological approach which does not assume a physicalist reference frame to 3D perception, and explain why, from a methodological standpoint, it better captures the nature of our 3D perception. In §4, I will outline a tripartite encoding framework and explain how this framework is more consistent with the phenomenology, psychophysics, neurophysiology and adaptive logic of 3D perception.

### (a) Phenomenology versus representationalism

When we look out into our visual world, we have conscious awareness of a 3D space inhabited at various locations by 3D surfaces and objects. The conventional textbook way to think about our perception of the 3D world is as something akin to peering out of a window onto an objective external reality: an objective reality that exists—*in the way that we see it*—independent of the observer. More specifically, the idea is that the visual system infers and reconstructs*,* through a process of inverse-optics, a unitary, veridical and internally consistent ‘representation’ of this objective external reality from the information available in the 2D optic array, and that it is this representation that we perceive.

This view is often explicit in the computational formulations underlying past and present models of the perception of 3D object shape and space, including models deriving from early work in computer vision (e.g. [1–3]) to the more recent dominant model of human 3D vision as a problem of probabilistic inference ([4,5]; see also various chapters in [6]). For example, the most prominent variant of the latter approach (maximum-likelihood estimation, MLE) explicitly assumes that each component of sensory information specifies unbiased (veridical) estimates of objective mind-independent properties such as distance, depth, slant, and 3D curvature, resulting in a unitary veridical representation of objective 3D structure [4,5]. Yet, the perceptual phenomenology of 3D space, along with related psychophysical observations, contradicts the idea that what is delivered by the visual system is an objective, unitary and internally consistent view of the external world.

But how do we define the phenomenology of 3D space? Which aspects of our perception of 3D space should we refer to as ‘phenomenological’? One way is to take the approach originating in the seminal work of Franz Brentano [7] which formed the groundwork for the development of *Gestalt* psychology, which in turn partly influenced researchers in spatial vision such as J. J. Gibson [8,9]. According to this phenomenological approach, *everything that is perceived*, whether objects, surfaces, colours, shapes, distances, space, depth, etc., constitutes phenomenology, and all aspects of these phenomenological entities and attributes have intentional content. Therefore, proper analysis or theorizing about such perceptual constructs must, as a starting point, begin with rigorous analysis of first-person introspections, rather than a search for mind–world correspondences [7].

The conventional viewpoint, aligned with inferential and representational approaches to perception, and much of analytic philosophy, is that perception consists of two forms of content. The most important forms are that aspect of perceptual content in which one can establish reference to objective properties in the external world, and another, purely subjective form of content called ‘qualia’. The term 'phenomenology', under this view, is typically associated with the latter and not the former. Phenomenology (qualia) is viewed as that part of perception that consists of subjective, non-functional, non-inferential, non-intentional mental content. Under this view, entities like perceived surfaces or objects, and associated perceived geometric attributes or properties (such as shape, depth, distance, curvature, etc.) are not quales or ‘phenomenological’, since they both specify content that is objective (they ‘represent’ the self-same objective entities and properties residing in the external world) and have functional and intentional content. Phenomenology (qualia) under the standard understanding is then simply that aspect of perception associated with subjective so-called ‘raw feels’: the redness of red, the bitterness of orange peel, the subjective feeling of pain, etc. (see [10]). According to the standard view, an analysis of phenomenology is not going to help us understand much about perception beyond the delimited subjective component of perception, so-called qualia. Thus, for a representationalist perception scientist or analytic philosopher, if one's aim is to understand the ‘representations’ underpinning perception, one must focus on the analysis of ‘objective’, functional components of perception (surface, shape, distance, depth, slant, what have you). The analysis of ‘subjective’ aspects of spatial perception (e.g. the ‘qualia’ of depth associated with stereoscopic vision) is of limited interest and has little bearing on the underlying representation of spatial entities, attributes and parameters.

I will argue that there are two problems with this approach. First, it dismisses the efficacy of the analysis of phenomenology and first-person introspective accounts in the scientific goal of determining the spatial encodings underlying 3D perception. Second, it creates an artificial dichotomy between putatively objective, functional content and putatively non-functional, non-intentional, subjective content (qualia/phenomenology).

My argument here follows the phenomenological approach. First, phenomenological analyses, including aspects that may appear to be ‘mere qualia’, are a crucial starting point to grounding theories and empirical investigation of perception. Second, *all of spatial perception* must be treated as phenomenological. Phenomenological analysis, therefore, becomes critical in uncovering the nature of the encodings underlying our perception of spatial entities, attributes and parameters. This view allows a more meaningful discussion of the relevance of ideas related to spatial perception, such as whether spatial encodings have anticipatory motor content constitutively embedded within them or whether related concepts in spatial perception, such as ‘affordance’ and ‘embodiment,’ can be suitably captured by a spatial encoding model at both a micro- and macro-perceptual level (a topic that will be covered in detail in §4).

In the next section, I will outline important aspects of phenomenology and psychophysical observations that gainsay the assumptions and claims of the prevailing inferential and representational model of 3D perception.

## 2. Phenomenology and psychophysics of 3D perception

### (a) Phenomenology and psychophysics of stereopsis and picture perception

**Figure 1.**
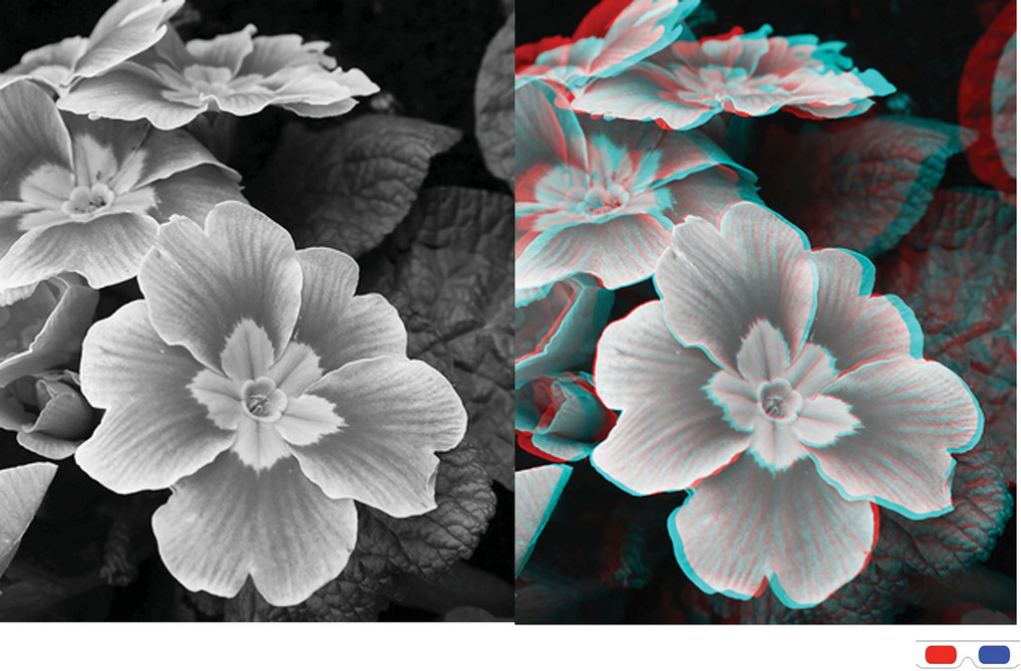
Left: a single picture of a 3D scene. Right: a stereoscopic anaglyph image of the same scene. This image should be viewed with red and blue/cyan tinted stereoscopic glasses (red on left, blue/cyan on right). Original images courtesy of Adrian Ston. Copyright © 2011 Adrian Ston.

Historically, the most widely discussed problem in the phenomenology of visual space is the characteristic impression of depth obtained in stereoscopic or real scenes in contrast to pictorial images (figure 1; [11–16]). While we obtain an impression of 3D shape and spatial layout in pictorial images (figure 1*a*) that matches in many ways what we perceive in the real version of the depicted scene, the impression of three-dimensionality lacks certain characteristic phenomenological impressions: object solidity, object tangibility (the feeling one can touch things), palpable negative space between objects and an overall sense of realness [11,13,14,16–18]. This is the impression that is typically associated with the term *stereopsis*. Similarly, while both monocular and binocular viewing of real scenes yields a similar perception of 3D object shape and layout, the impression of depth separation under binocular viewing is more compelling [19]. The compelling impression of depth separation in real scenes under binocular viewing, however, diminishes rapidly for farther viewing distances even for very large magnitudes of depth separation [20–22]. This can be observed by judging the difference in depth impression between one-eye and two-eye viewing of a real scene. A large difference in depth impression is evident in near space, but the difference—even for very large inter-object distances—reduces rapidly with viewing distance [18]. Picture viewing provides an additional complication in that the observer has a phenomenology where they simultaneously perceive both a virtual pictorial space within the image and the real tangible surface of the picture itself [19,23,24].

Inferential models have long neglected these important phenomenological observations and are unable to provide satisfactory explanations of them. However, a few explicit or implicit arguments have been put forward to explain the difference in phenomenological impression under different conditions where we perceive three-dimensionality, relying on some aspects of the inferential or direct-perception standpoints (e.g. [11,25,26]).^1^

One class of arguments for the phenomenological difference between pictorial depth and depth in real scenes under binocular viewing (stereopsis) rests on the idea of a fundamental difference between the visual depth cues of binocular disparity, motion parallax and ocular convergence on one hand, and so-called pictorial depth cues (e.g. perspective, shading, texture, etc.) on the other, a distinction first drawn by von Helmholtz [27] and later appearing in various iterations in the literature (e.g. [11,28]). Other variants distinguish between primary (convergence, accommodation, binocular disparity) and secondary (pictorial depth cues and motion parallax) depth cues (see [8,29]). The compelling depth impression obtained in real scenes or stereoscopic images (stereopsis)—but not pictures—is then attributed to the claim that only the primary depth cues can lead to a perceptual experience of depth (e.g. [17,30]). But this view is challenged by the fact that a compelling impression of depth similar to binocular stereopsis can be obtained where none of the so-called primary cues (vergence, accommodation, disparity), nor motion parallax, is consistent with the perceived 3D scene; and where the only cues specifying depth are ‘secondary’ or pictorial, specifically under synoptic^2^ or monocular-aperture^3^ viewing of single pictures [11–14,32,33].

A related argument distinguishing pictorial and real depth perception claims that depth perception in real or stereoscopic scenes is the perception of ‘quantitative’ depth, while pictorial depth is simply a ‘qualitative’ cognitive inference based on pictorial cues, that might, for example, be based on learning from prior perceptual experience of depth from more direct perceptual cues such as binocular disparity ([30,34]; see also [25])*.* But this argument is challenged by a wide range of psychophysical results that show that reliable quantitative judgements of 3D structure can be made when viewing single pictures monocularly or binocularly (e.g. [26,31,32,35,36]). It also goes against obdurateness, imperviousness to learning and the automaticity of pictorial 3D perception based on even the simplest of visual cues. Even in cases where there can be bi- or multi-stability in pictorial percepts, the allowable states of the multi-stability are strictly deterministic on the nature of the underlying visual input and immune to cognitive influence. Finally, another problem with this view is that it implies that during the perception of monocular stereopsis in a single picture (when viewing with a synopter or monocular aperture), the very same cues that enable merely a cognitive inference of pictorial depth at one instant (normal picture viewing) are somehow transformed to yield a non-cognitive perceptual experience of depth in another (the impression of stereopsis).

Another class of explanations regarding the difference in phenomenological impression between pictures and real or stereoscopic scenes argue that depth in pictures appears less compelling because the observer is simply perceiving ‘less’ depth owing to the ‘flattening’ effect of binocular disparity. When viewing a picture with both eyes, binocular disparity signals the flat surface of the picture, and contradicts the depth specified by pictorial cues (perspective, shading, texture etc.). The phenomenological enhancement of depth impression in single pictures known as monocular stereopsis (e.g. synoptic viewing, monocular aperture viewing) is ascribed to an increase in perceived magnitude of depth owing to the removal of the conflicting disparity cue [11,12]. This idea is consistent with probabilistic cue-integration models that propose a linear weighted combination of individual cue estimates to derive 3D parameters [5]. But the explanation can be discounted by a range of psychophysical evidence that reveals no quantitative change in any aspect of perceived depth (depth separation, slant, 3D dihedral angle, 3D curvature) comparing binocular and monocular viewing of pictures ([31,32,35,36]; cf. [26])^4^, including conditions where monocular stereopsis is perceived [32]. Moreover, eliminating disparity alone by closing an eye is insufficient to generate an impression of monocular stereopsis in pictures [32], suggesting that the removal of disparity is not by itself the determining factor for the enhancement in depth impression [18]. Finally, pictorial depth itself has been found to not follow the statistical optimality proposed by the MLE model [37], weakening arguments applying this framework to explain monocular stereopsis.

In terms of the diminishment of the strength of the phenomenological impression of depth separation at greater viewing distances, inferential models often ascribe it to the fact that binocular disparities rapidly reduce in size with viewing distance (e.g. [20]). However, this explanation contradicts the main assumption regarding depth from disparities in these models. According to these models, what is ‘perceived’ as depth are not the retinal disparities themselves, but the depth values derived from scaling disparities with egocentric distance cues (e.g. [4,20,38]). Disparity detection is accurate and precise for even very small disparities, and egocentric distance perception is accurate to at least 25 m [39], suggesting there should be no noticeable decrease in disparity-derived depth at these distances. Yet, the phenomenal impression of depth separation (stereopsis) shows marked reduction at even these distances [18].

### (b) Phenomenology and psychophysics of surface shape perception

**Figure 2.**
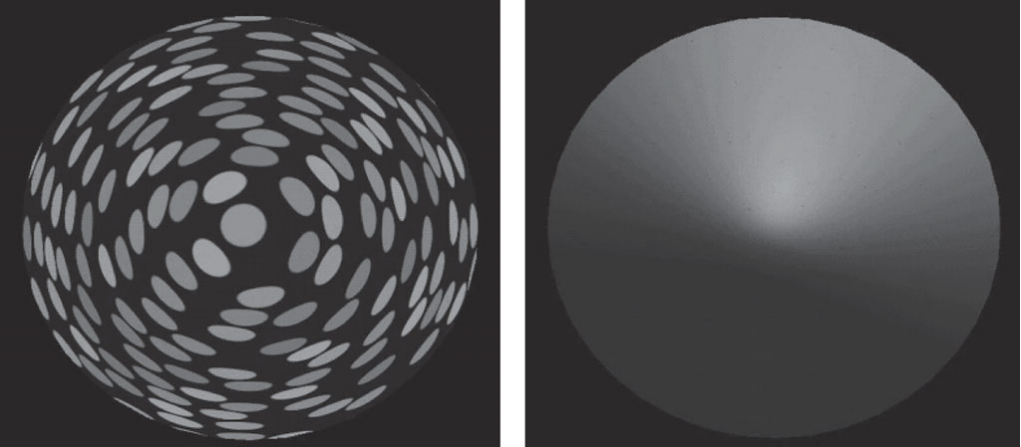
A parabolic cone rendered with either a texture pattern (left) or shading pattern (right). The modelled geometry of the two cones (ground truth) is identical, yet the phenomenology is that the left cone appears deeper overall, while the right cone appears shallower, but with a more pointed tip, something that is confirmed by psychophysics [40]. (Image courtesy of F. Domini.)

Inferential models such as the MLE model derive their logic from the assumption that every depth cue delivers on average an unbiased (accurate) estimate of 3D properties [4,5]. But there is clear evidence that perceived object shape deriving from the same ground truth but based on different cues is perceived with significantly different depth and 3D curvature, as experienced phenomenologically and measured psychophysically ([40], figure 2; see also [41]). Similarly, the assumption of the accuracy (unbiasedness) of cues is contradicted, for example, by psychophysical evidence showing systematic underestimation and overestimation of depth from binocular disparity depending on small differences in viewing distance [38,42]. Explaining these findings under the MLE model usually requires the introduction of ad hoc variables such as a ‘flatness prior’. Probabilistic inferential models, such as MLE, also imply an intrinsic variability (Gaussian distributed) in the perception of depth properties, suggesting that one should experience slightly different perceptions of object shape from moment to moment (or trial to trial) when exposed to the very same stimulation. Our phenomenological experience, however, contradicts this: we do not have a sense of uncertainty about 3D object shape in the real world regardless of which cues specify object shape. For example, in figure 2, it is not as though there is greater uncertainty in the impression of 3D shape for the right shape compared with the left shape as is predicted by MLE models. The right shape is specified by shading, which is considered a more variable cue to depth (larger just noticeable differences, JNDs) than the texture cue specifying the left shape; yet the main discernible perceptual difference is that the 3D shape on the left appears deeper, not that there is more phenomenologically uncertainty in the impression of 3D shape on the right.

While these phenomenological and psychophysical observations cannot be modelled with prevailing inferential models such as MLE, a more recent quantitative model described in this issue [41] that rejects assumptions of inference, objectivity, veridicality and statistical variability in perceptual attributes, is able to successfully account for them.

### (c) Psychophysics and phenomenology of egocentric and exocentric distance at the ambulatory scale

An important explanatory challenge to inferential models already described above is the diminishment in the phenomenological impression of exocentric depth separation between objects (at increasing distance from the observer), even though the phenomenological impression of the distances to objects (egocentric distance) does not appear to similarly diminish with distance from the observer. Empirical findings have long confirmed this dissociation. Human observers can accurately estimate the egocentric distance to an object up to least 25 m using a blind walking paradigm [39]. However, in studies where observers were asked to judge both the egocentric distance to and exocentric distance between two separate objects, results show accurate estimation of egocentric distances (using a blind walking paradigm) but significant inaccuracy (underestimation) of the exocentric distance between the same two objects when matching to an adjustable frontoparallel interval. This finding has been confirmed for distances ranging from 2 to 15 m [39,43]. A related phenomenological aspect of depth perception at ambulatory distances is the fact that equidistant intervals in the depth (sagittal) plane appear progressively foreshortened (nonlinearly) with increasing distance [44,45]. This effect is most readily apparent phenomenologically when viewing dashed lines dividing a roadway. Moreover, there is the phenomenological impression, as one walks along the road, of the dashed lines ‘growing’ or ‘stretching’ in length as one comes closer to each interval [46]. While the finding that egocentric distance perception is accurate over a wide range of distances is consistent with inferential models (which assume the derivation of a veridical representation of space), the finding of the dissociation between egocentric distance and exocentric (inter-object) distance or interval perception, along with other associated phenomenology, cannot be accommodated in such models.

We have seen above important phenomenological observations in depth, distance and 3D surface shape perception, matching psychophysical data, that are wholly inconsistent with the basic assumptions of the prevailing representational and inferential model of 3D perception. In the next section, I will outline and justify the alternative conceptual and methodological approach that starts with phenomenology and eschews the main assumptions of the inferential approach.

## 3. An alternative conceptual and methodological approach

The observations in the previous section point to an alternative approach to the study of 3D perception that makes the following claims: (1) an understanding of human 3D vision remains incomplete without systematic consideration of phenomenology; (2) the perception of a 3D world does not arise from a ‘reconstruction’ or ‘inference’ of an objective external reality; and (3) the perception of objects and space is underwritten by multiple often internally inconsistent encodings optimized to specific spatial and behavioural constraints, function and region of operation.

The critical aspect of the alternative approach lies in the idea that the properties and entities that define the perception of 3D objects and space are neither wholly ‘subjective’ nor ‘objective’ but constitute *relational content* (see [47,48]). More specifically, the contents and structure of the spatial encodings are bound to the peculiarities of the relationship between the nature of sensory sampling and motor capacities of the agent on one hand and the underlying physical substrate on the other. The properties, attributes and entities that make up our perception of 3D space are therefore not referents to objective physical properties, attributes or entities that exist independent of the agent's apparatus. Rather, the encodings are a ‘plan’ about how ‘what is out there’ can be interacted with, by the agent, through motoric and mental operations. They specify the conditions and constraints available to behaviour given the sensorimotor competencies of the agent, resulting in the visual awareness of a ‘space of operation’ coded in idiothetic or proprioceptive units or terms (most broadly construed) rather than a ‘view of an objective representation of reality’. Spatial encodings are fundamentally observer-relative and have a constitutively embedded content of motor anticipation and agency. 3D visual perception can thus be regarded as a sort of proprioceptive sense: a mental grasping of objects and space in terms of the agent's own internal mental and motor capacities.

These views are sympathetic with aspects of proposals from other researchers. The idea of distinct spatial encodings for perception has, for example, been suggested in Zimmerman *et al*. [49] and Loomis *et al*. [43]. The idea of *relational content* is championed in Turvey [47] and Warren [48] arguing against the subjective/objective content dichotomy implicit in representationalist approaches. The idea that spatial attributes have constitutively embedded content related to motor anticipation is partially compatible with Gibson's notion of affordances [9] and Maturana & Varela's concepts of embodiment in perception [50,51].^5^

Importantly, there is sound evolutionary logic to the idea that the encoding of space is not a reconstruction of an objective reality but one where the primary attributes and entities of space themselves (distance, direction, surface, object shape) are agent-contingent.

### (a) Evolutionary arguments supporting the phenomenological approach

**Figure 3.**
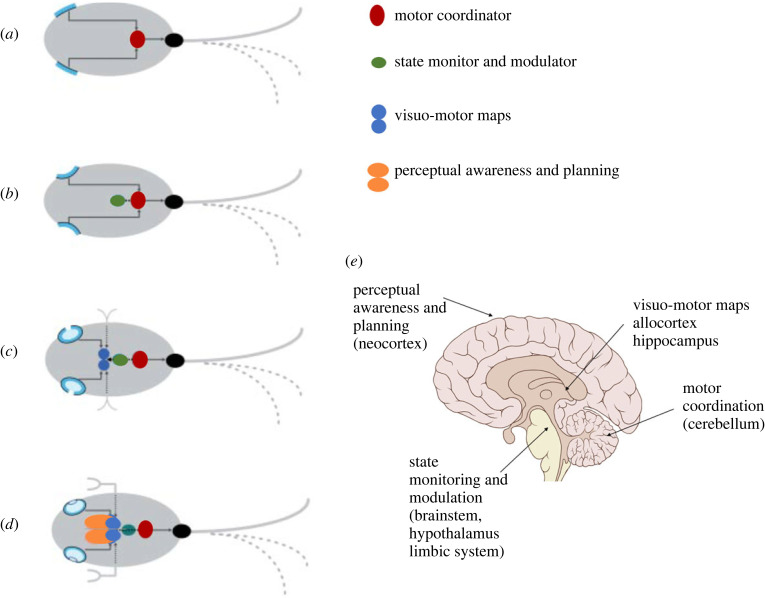
(*a–d*) Hypothetical stages of the evolution of an animal perceptual system. (*a*) A simple organism with a reflexive light-based behavioural response. The light-sensitive photoreceptor sheets are linked to its locomotor apparatus (tail). The red oval represents a neural ‘visuo-motor coordinator’ and the black oval the ‘motor plant’. (*b*) A slightly more advanced organism which has evolved neural structures (green oval) that modulate light-mediated locomotion based on monitoring current adaptive needs (e.g. satiety, arousal, safety). (*c*) A still more advanced organism with a complex neural ‘map’ relating light patterns, locomotion, past behaviour and adaptive consequences (blue circles). (*d*) A sophisticated ‘perceiving’ organism with an advanced eye and tactile sensors. The organism has evolved the neural apparatus (yellow ovals) to consciously monitor, in real time, the information content of the complex neural ‘maps’ (blue ovals) in order to plan behaviour. It is this information that the organism perceives in visual awareness, rather than a representation of an objective ‘external world’. (*e*) Mammalian (human) brain structure reflects the basic division of function predicated by the hypothetical stages of the evolution of perceptual systems. Figure adapted from [54].

Consider the plausible stages of evolution of visually guided organisms (figure 3)—from an organism with simple reflexive light-mediated responses to animals with conscious visual awareness of an external world (such as humans). It is hard to fathom an adaptive logic for the evolution of independent neural mechanisms geared towards deriving an objective ‘inference’ of an external 3D world from a retinal image (so-called inverse optics). It seems more plausible that the development of neural information content at each stage entailed increasingly sophisticated linkages between the pattern of activation of the light sensing array and adaptive motor behaviour. It is this linkage that constitutes the content of the spatial encoding in the neural substrate of visual system.

For example, if we consider the earliest stage (figure 3*a*), the ‘information content’ in the rudimentary neural apparatus (motor coordinator) generated from the activation pattern on the photoreceptor sheets due to light impinging from a particular direction is essentially the ‘direction and amount by which the tail must flip for an adaptive response’, rather than a quantitative geometric parameter specifying the ‘direction of impinging light’.

The behavioural competencies of the agent are thus constitutively embedded in the phylogenetically evolving spatial encoding. When conscious perceptual awareness of visual space emerges at later stages of evolution (figure 3*d*), what the agent is ‘aware of’ is therefore not an objective ‘representation’ of the external world, but rather an awareness of the peculiar and complex neural information content linking behavioural capacity with the unknown underlying physical substrate of the external world.

### (b) Empirical study of perceptual 3D space (psychophysics and phenomenology)

Given the alternative view of the information content of visual awareness, how should we approach the scientific study of human perception of three-dimensionality? The first step is to distinguish between the inferentialist, physicalist approach (aiming to understanding how *mind-independent* properties are represented) and the phenomenological approach (aiming to understanding the patterns in the *mind-dependent* appearance of things).

The physicalist approach to 3D perception relies entirely on psychophysical investigations of the correlation between physical stimulation and quantitative perceptual judgements (or neural activation) based on a model of perceptual space derived from our geometric understanding of the external world as specified by classical physics. The phenomenological approach, instead, seeks to first develop a model of perceptual space from an analysis of phenomenology alone, independent of any assumptions from our geometric or physicalist understanding of the external world. The crucial difference between the two is that in the physicalist approach, an underlying model of space is pre-determined based on classical physics and geometry, while in the phenomenological approach, the model is itself derived from analysis and understanding of phenomenology.

Note that both these approaches are dependent on perceptual phenomenology since even psychophysics relies on judgements that derive ultimately from how we perceive things (phenomenology). However, the distinction is that the inferential approach only considers those aspects of phenomenology where there is a ready *quantitative* operationalization of the phenomenological construct (e.g. distance, slant) that can be aligned with a corresponding physical geometric property. Other so-called ‘qualitative’ aspects of phenomenology, e.g. the experiential appearance of stereopsis (tangibility, negative space, realness), or the anticipatory phenomenology in distance or depth perception, are ignored because they are deemed unquantifiable or irrelevant to understanding the underlying encoding, and do not fit the pre-defined physical model of space. By contrast, in the phenomenological approach, these so-called ‘qualitative’ phenomenological aspects are important clues to how the information content of the neural encoding underlying perception is structured and what its adaptive significance is. The phenomenological approach is, however, in agreement with the physicalist approach in that it assumes cross-observer objectivity. It also assumes that the validation of any theory or model ultimately requires conducting psychophysical or neurophysiological investigations for falsification and replicability of predictions arising from the model developed from phenomenological considerations.

The best example historically of the scientific consequences of these two approaches (physicalist versus phenomenologist) is in colour perception.^6^ The earliest models of colour perception embraced a physicalist approach: attempting to model colour perception by observing the behaviour of the physical substrates of colour (pigments or coloured lights). This approach led to, among other things: (1) Newton's enumerating seven perceptual primary colours based on what he saw as the distinguishable colours of the physical light spectrum; (2) the establishment of the idea of three primary colours and trichromacy theory based on observing hue mixing either in physical pigments (red, yellow, blue; [55]) or light (red, green, blue/violet; [27]); and (3) unsuccessful attempts to develop a workable perceptual trichromatic colour-mixing model based on modelling observations in physical light mixing (see [57]). While trichromacy theory correctly presaged the existence of tri-channel physical wavelength transduction at the retinal cones [58], its main perceptual prediction that trichromatic units should represent three perceptual primary colours (red, green, blue) was empirically invalidated since their peak response occurs at wavelengths that appear greenish-yellow, green and violet (see [55]). Moreover, the trichromatic model failed to satisfactorily account for many critical aspects of colour perception, including: the phenomenological purity of yellow (which under trichromacy is considered a colour mixture); the specificity of the colour inducer–afterimage pairings (e.g. blue yields a yellow afterimage); the phenomenological absence of certain colour mixtures (e.g. red with green); paired colour loss in colour-blindness (e.g. deuteranopes perceive blue/yellow distinctions but not red/green distinctions, even though yellow is claimed to be a mixture of red and green according to trichromacy theory); phenomenological colour differences in saturation/desaturation, lightness/darkness and whiteness/blackness; and the existence of colours like olive green that cannot be generated simply by decontextualized light mixing (see [55]).

By contrast, Hering's phenomenological approach [56], in developing the opponent colour model, provided a framework where all these critical observations can be systematically and explicitly accounted for. An important point to note is that the opponent processing theory of colour requires no understanding of (or assumptions based on) the wavelength property of light or even of physical pigment or coloured-light mixing. It is an abstract hypothesis based on phenomenology alone, entirely independent of any understanding or classification of physical properties. Even Hering's critical insight of simple antagonistic neural mechanisms underlying colour opponency required no previous knowledge of physical or biological substrates. Indeed, at the time, the neuronal doctrine itself had not yet been established, and the idea of antagonistic or inhibitory neural mechanisms was not known [55]. The opponent process model and the proposed neural mechanism were eventually validated psychophysically in Hurvich & Jameson’s hue-cancellation experiment [59] as well as with the discovery of opponent cells in lateral geniculate nucleus [60]. Importantly, in the applied domain, the most widely used device-independent colour spaces that best model human colour experience, e.g. in terms of perceptual uniformity, are derived, in whole or part, from the opponent model (CIELab, NCS, Munsell). The historical account of colour perception research demonstrates that perceptual models that rely on modelling perceptual space on the basis of physical observations alone will invariably fail to fully explain human perception.

### (c) Modelling the phenomenology of 3D space

The main aim of the dominant approach to 3D perception (probabilistic inference) is to determine how objective mind-independent 3D structure can be inferred in a bottom-up manner from visual stimulation. The task becomes identifying and enumerating visual ‘cues’ and developing a quantitative model that explains how 3D structure is ‘inferred’ or directly specified by these cues individually or in combination [5].

**Figure 4.**
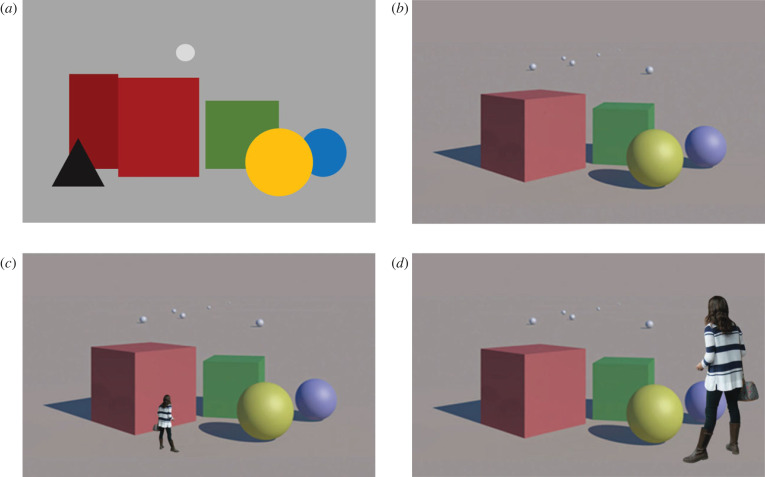
(*a*) Perception of depth order only. The perception of a visual field where regions or two-dimensional patches are perceived as ordered in depth with respect to the viewer. (*b*) Perception of unscaled depth. The 3D shape and layout of objects is perceived but the scale of the objects and scene is ambiguous. (*c*,*d*) The same image as in (*b*), highlighting the scale ambiguity of pictorial space perception, which can be subject to different cognitive interpretations of scale based on familiar-size information. Image of human figure in (*c*,*d*) courtesy of www.escalalatina.com licensed under the Creative Commons Attribution 4.0 International License.

**Figure 5.**
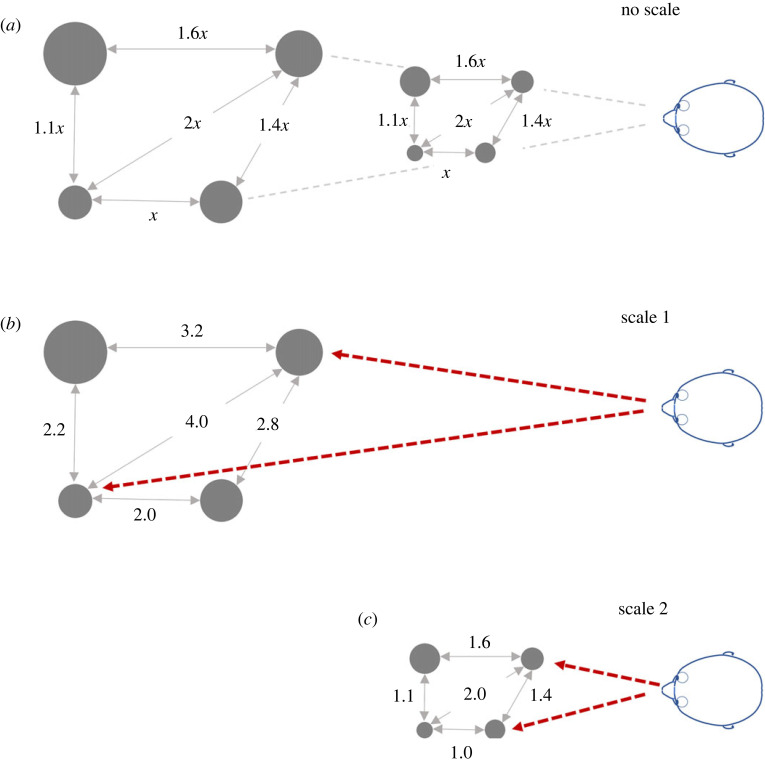
Psychophysical operationalization of the perception of unscaled depth, scaled depth (exocentric distance) and egocentric distance. (*a*) Diagrammatic representation of unscaled (relative) depth perception (perception of 3D object layout and shape). The scale of the objects and scene is unspecified. The observer perceives a specific layout (shape) in terms of ratios of distances, but cannot distinguish between the two configurations. The observer does not perceive the egocentric distances of the objects or the actual spatial separations between them. (*b*,*c*) Two examples of cases where the observer perceives the spatial scale and the exocentric distances among the objects. Assuming an internally consistent representation, this implies that the observer is also aware of the egocentric distances of objects (red dashed lines).

The alternative phenomenological approach does not assume that the 3D world that we perceive is mind-independent. Therefore, the starting point for the modelling is the higher-order first-person phenomenological description of the entities and attributes that make up 3D space. In terms of entities, as human observers, we perceive surfaces and discrete objects that are arrayed in a visual space before us. We can then distinguish among four distinct perceptual or phenomenological modes of the spatial experience of these entities:

(1) The perception of a visual field as partitioned into discrete entities ordered in depth with respect to the observer (figure 4*a*).
(2) The perception of the 3D shape and layout of surfaces and objects (orientation, slant, surface shape, inter-object layout, etc.). In psychophysical terms, this implies the encoding of unscaled depth relations (distance ratios, figures 4*b* and 5*a*).
(3) The perception of objects (and the space between them) as having a specific spatial extent or scale. In psychophysical terms, this implies the encoding of exocentric distances (scaled depth) within and among objects (figure 5*b*,*c*).
(4) The perception of an object as being located at a particular distance from the observer. In psychophysical terms, this implies the encoding of the scaled egocentric distance (figure 5*b*,*c*, red dashed lines).

Standard inferential models (like MLE) typically do not make a fundamental distinction among these different modes of spatial experience (though see [49,61]). Instead, the implicit assumption in these models is that the visual system infers a master representation of the scene where, in effect, the egocentric coordinates of all the points making up the surfaces and objects in the scene are explicitly or implicitly specified as a SLAM^7^-like or constructive solid geometry representation. The four modes of perceiving listed above are then simply derivative aspects of this master representation, For example, a map of the egocentric coordinates of all points in the scene directly entails knowledge of the egocentric distance of points (4) and the depth order of points with respect to the observer (1); the encoding of egocentric coordinates also entails knowledge of the exocentric distances between points or objects, and the scale of the objects (3), which, in turn, entails knowledge of the ratio of distances between objects and therefore their 3D layout and shape (2).

But as we have already seen in §2, phenomenological and psychophysical observations question the view of a single master representation of space and instead support the view that these different modes of spatial experience are underwritten by distinct and dissociable encodings.

While the prevailing scientific models of 3D perception have neglected the consideration of phenomenology, there have been efforts in the philosophy of perception literature to address phenomenological aspects of space perception. For example, Tye [10] discussed the phenomenology of binocular stereopsis as something that made perceived depth more ‘definitive’ in comparison with pictorial depth. Matthen [63] attributed the perceptual feeling that one is viewing a real rather than a pictorial scene to the ability to perceive egocentric distance in real scenes but not in pictures, with the dissociation linked to the dual visual stream account [64]. Hibbard [65] associated the impression of binocular stereopsis to dorsal stream representations. Related proposals on differences in spatial perception in relation to pictures, real scenes and *tromp l'oeil* are further analysed in Nanay [66] and Ferretti [67].

In addition to a dissociation in spatial encodings predicated on the four phenomenal modes of spatial perception outlined above, it is also important to distinguish among the different regions of space in which these modes may be most applicable. Cutting & Vishton [68] provided a very useful distinction of visual space based on both functional considerations and availability of visual signals, distinguishing between personal space (within 2 m of the observer), action space (between 2 and 30 m from the observer) and vista space (greater than 30 m from the observer). In the rest of this paper, I will broadly follow this distinction in arguing for differences in the optimal regions of operation of the different encodings.

## 4. A tripartite model of 3D spatial perception

**Figure 6.**
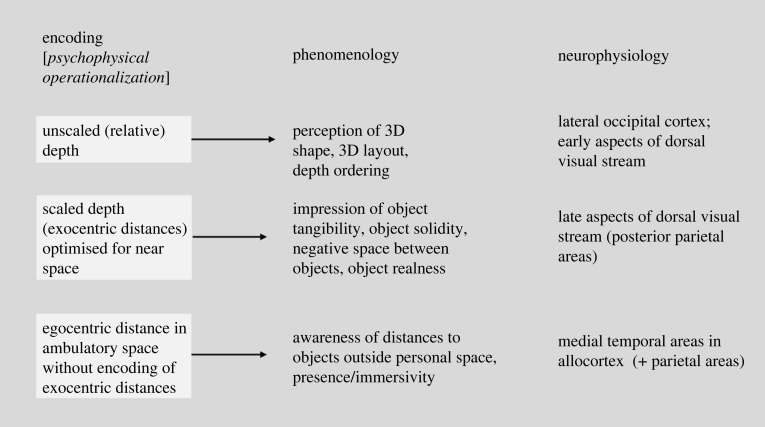
Diagrammatic overview of tripartite encoding.

In this section, I will outline a new model of spatial perception that conjectures a tripartite dissociation among encodings of unscaled (relative) depth, exocentric distance (scaled depth) and egocentric distance (figure 6). I argue that this tripartite distinction is able to account for a wide range of psychophysical and phenomenological observations and additionally is an evolutionarily plausible model of 3D spatial perception. It derives from a prior proposal [18,19]) that argued for bipartite dissociation between encodings of exocentric distances (scaled depth) and relative (unscaled) depth that developed from an analysis of the phenomenology of stereopsis and associated empirical findings, which I address below first. I then motivate the conjecture that, counterintuitively, the encodings of exocentric distance (scaled depth) and egocentric distance are also dissociated, at least in ambulatory space.

### (a) Dissociation between the perception of unscaled depth and scaled depth (exocentric distance)

Analysis of the phenomenological difference between the perception of depth in pictorial images (pictorial depth) and the perception of depth in real scenes under binocular viewing (stereopsis) led to the hypothesis that the awareness of visual space is underwritten by at least two distinct spatial encodings [18,19]: one underlying the awareness of object layout and shape (unscaled depth) and the other underlying the awareness of spatial scale and exocentric distances (scaled depth). The former encoding was conjectured to underlie the perception of pictorial depth in which we perceive 3D shape and layout but where the scale of objects is ambiguous (figure 4*c,d*). The encoding of spatial scale and exocentric distances (scaled depth) is hypothesized to underlie the phenomenological impression of object solidity, tangibility, and impression of negative space between objects, which give rise to the overall impression of perceptual ‘realness’ associated with stereopsis.

This hypothesis provides a unified basis for understanding a variety of important observations in the phenomenology of space in both pictures and real scenes. For example, it explains the visual duality in picture perception, where there is both a perception of a virtual unscaled 3D pictorial space and the simultaneous awareness of a real tangible picture surface [19]. It also accommodates the observation that the impression of stereopsis can be obtained in conditions where neither binocular disparity nor motion parallax is available (synopter, monocular aperture), since the hypothesis aligns the phenomenological impressions of stereopsis and realness with a type of spatial encoding (scaled depth) rather than specific depth cues.

The linkage between the phenomenology of stereopsis and a scaled depth encoding also provides a more satisfying account of the specific characteristics of this phenomenology. Since specification of spatial parameters (such as exocentric depth) in terms of body scale is necessary for visual guidance of manual action, it makes sense that the phenomenology associated with it is characterized by an impression of object solidity and tangibility (the feeling that you can reach out and touch or grasp something). The impression of depth associated with stereopsis can therefore be said to constitute a direct awareness of the ‘capacity for motor interaction’ [13], something that is lacking under normal viewing of objects in pictorial space. In support of this, empirical evidence has demonstrated that observers are able to discriminate (with a manual response of their unseen hand) the position of objects in depth depicted in single pictures only when the impression of stereopsis is induced (monocular stereoscopy) and are unable to do so when the same images are presented under conditions where monocular stereopsis is not induced [69].

A further conjecture of the dual encoding hypothesis [18,19] was that the strength of the phenomenological impression of stereopsis (objects' solidity, tangibility, negative spatial separation, etc.) is linked to the statistical reliability with which the scaled depth separations are specified, depending on the specific stimulus and viewing conditions. This entails that the impression of stereopsis or ‘realness’ lies on a continuum, where the strongest impression is predicted to occur within the personal space of the human observer (under 2 m) and degrades with distance such that at very far distances (vista space) there is no impression of stereopsis, and objects perceptually appear almost pictorial. This is supported by empirical evidence and consistent with modelling the reliability of scaled depth with viewing distance based on known variability of perceptual estimates from visual depth and distance cues [18]. The proposed link between the phenomenology of stereopsis and the statistical reliability of scaled depth estimates can also potentially account for variations in the strength of the impression of stereopsis under various modes of viewing pictorial images (e.g. stereoscopy, synoptic viewing, monocular aperture viewing, addition of depth of focus blur; see [18]).

However, a more recent model of 3D cue integration by Domini [41] rejects the notion of the presence of statistical noise in perceptual estimates deriving from depth cues in the manner proposed by the prevailing probabilistic model of cue integration [5]. This new conjecture suggests an alternative account, where the variation in phenomenological strength of the impression of stereopsis is directly linked to the derived *magnitude* (gain) of scaled depth separation rather than the statistical reliability of a derived magnitude. Under this interpretation, the loss in strength of the impression of stereopsis with increasing distance of the objects in real scenes is linked to an exponential reduction in the actual derived magnitudes of exocentric distance (scaled depth separation). For example, the exocentric distance between two objects 50 m away separated by (say) 20 m is encoded as being less in absolute terms than the separation between two objects 5 m away separated by (say) only 2 m. This interpretation is more compatible with the idea that the scaled depth encoding is an adaptation that evolved specifically for personal (or reach) space and only present in residual and non-adaptive form in regions that are beyond the zone of operation of the specific motor actions it facilitates (e.g. grasping, manipulation). The underlying assumptions of Domini's proposal are also more compatible with the proposal put forward in this paper.

### (b) Dissociation between the perception of egocentric and exocentric distance

According to the standard inferential models, an accurate inference of the egocentric distances to two objects (figure 5*b*,*c*) automatically entails an accurate inference of the exocentric (inter-object) distances as a simple numerical subtraction. However, as previously mentioned, the phenomenological introspection of egocentric and exocentric distance perception beyond personal space (greater than 2 m) reveals a clear dissociation, where the impression of exocentric depth separation appears to rapidly diminish with increasing distance (even for very large depth separations), while the phenomenological impression of distance to objects does not diminish in the same way. Moreover, closing one eye when viewing a real scene substantially diminishes the impression of spatial separation between objects within action space, but the perception of the distance to an object appears unchanged (see [18,46]). The psychophysical data by Loomis and collaborators [39,43] described in the Introduction confirms this phenomenological dissociation. The data [39,43] show that the underestimation of inter-object distance increases significantly with the egocentric distance to the objects. It also shows that egocentric distance estimates are comparable under monocular and binocular viewing, suggesting no role of binocular disparity on egocentric distance perception at the tested distances. This finding has also been replicated in a more recent study that tested distances up to 7 m [70]. However, exocentric distance perception judgements show significantly greater underestimation under monocular viewing, implicating the important role of disparity in the perception of exocentric (inter-object) distance—for example, deficits in grasping in observers with typical binocular vision [71].

This is further bolstered by findings that strabismic observers, who lack functional binocular vision and so do not obtain the phenomenological impression of spatial separation associated with stereopsis [17], are, however, able to judge egocentric distances of objects beyond personal space (3–7 m) with an accuracy comparable to individuals with typically developed binocular vision [70].

The dissociation between egocentric distance and exocentric distance perception, both phenomenologically and psychophysically, is observed for distances beyond personal space (greater than 2 m). But egocentric and exocentric distance perception appears to be interlinked within personal space, the region within which binocular disparity appears to be functionally optimized. For example, strabismic observers, who are unable to perceive depth from disparity, and lack the phenomenology of spatial separation associated stereopsis, show significant deficits on tasks that require egocentric distance judgement within reach space [72,73]. This is echoed in the data from observers with neurotypical binocular vision who show deficits in tasks requiring judgement of object egocentric distance under monocular viewing (but not binocular viewing) in reach space [71,74,75].

These results suggest that at least within reach space (<1 m), and potentially within personal space (<2 m), perception of egocentric distance might be achieved with the same encoding as that which underlies the perception of exocentric distances (scaled depth) and is hypothesized to underlie the characteristic impression of stereopsis (object solidity and tangibility, negative spatial separation, realness), both relying strongly on binocular vision. However, there is also evidence of a dissociation between exocentric and egocentric distance perception in near space [42], which suggests that more research may be neccessary to establish or reject a dissociation between egocentric and exocentric distances in reach space.

In contrast, perception of egocentric distances beyond personal space appears to be underwritten by a separate encoding that does not specify exocentric distances and does not depend on binocular vision, though it must be noted that the empirical record on dissociations in judgements between exocentric and egocentric distance in locomotor space is complicated. An excellent recent review of this work is by Warren [48]. For example, while visuomotor egocentric responses are accurate, verbal responses are underestimated (e.g. [76]). However, verbal reports draw on an additional cognitive element to consciously convert a perceived distance to an arbitrary scale which may itself systematically bias responses in a way that does not directly bear on what is experienced phenomenologically in terms of distances or depth. For the purposes of the arguments here, therefore, I restrict myself to interpretation of only direct visuomotor (e.g. walking) or perceptual (visual matching) judgements. Two further findings in this regard appear to complicate the idea of dissociation in encodings underlying (accurate) egocentric distance perception and (underestimated) exocentric distance judgements.

The first is that walking up to an unmarked location in front of one of two targets separated in the frontal plane, such that the egocentric distance to the main target is equal to the distance between the two targets (the observer and targets form an equilateral L), indicates that the egocentric distance is underestimated since the observer stops at a point specifying a larger physical distance from observer to the main target than the separation between the two targets [77]. This suggests that even egocentric distance is underestimated in some instances. Second, egocentric bisection, where an observer sets a marker to bisect the distance between the observer and a farther target, is accurate to large distances in open fields (e.g. [78]). This suggests that exocentric distances (between the marker and far target) are accurately perceived along with egocentric distance (between the observer and marker). Warren [48] has provided a very persuasive explanation of how these findings and the original Loomis* et al.* [39,43] findings can be reconciled based on proposing a dissociation between accurate perception of frontoparallel extents, which is said to rely on the horizon ratio (in action space and beyond) and underestimated egocentric and exocentric distance perception, which is due to an intrinsic bias to overestimate declination angle. This dissociation between the perception of frontoparallel extents and distances along the sagittal plane is also observed in work comparing distance and frontoparallel spatial extent perception in virtual reality and real scenes ([79]; see also [80]), though seminal work of Warren and collaborators on affordances also showed that frontal extents (measured as a function of passability) could be altered by changing the visually specified eye height [81].

Notwithstanding a potential dissociation between the perception of frontal and sagittal extents, there remains a phenomenological argument that aligns the two findings described above (equilateral L task, and egocentric bisection task) with the specific claim here of a fundamental dissociation between encodings for egocentric and exocentric distance perception. In the first case (equilateral L), the judgement, which appears to be an egocentric judgement, may in fact phenomenologically be a comparison of exocentric extents (the sagittal extent between observer and main target and the frontal extent between the two targets), where the sagittal extents are underestimated in comparison to frontal extents (as found in [39] and also found in Geuss *et al*. [79]). In the second example (bisection task), what appears to be a comparison of egocentric and exocentric sagittal extents (egocentric: the distance from the observer to the bisecting marker; exocentric: the spatial extent between the marker and far target) may in in fact rely on attending to and comparing, successively, the egocentric distances to the target and the bisecting marker. Thus, these findings are not necessarily in conflict with a proposed dissociation in encodings of (accurate) egocentric distance perception and (underestimated) sagittal exocentric distance judgement in action space and beyond. The phenomenological difference in perceiving or judging the distance *to* an object compared with judging the sagittal extent *between* two objects (even if one of those objects is the observer) may be a clue to supporting these arguments, and (at least in this author's mind!) informal observation appears to confirm this.

Further insights by Warren [48] on how certain motoric judgements appear to be susceptible to visuomotor learning (and after-effects) while certain perceptual judgements are not can also potentially be accommodated in the current account, though ultimately only further empirical study will reconcile these two views or favour one over the other.

### (c) Tripartite encoding of 3D space

Taken together, the observations of the previous two subsections imply that the encodings of object shape and layout, exocentric distances (scaled depth) and egocentric distances at the ambulatory scale are each distinct, pointing to a tripartite encoding of visual space [46]. The phenomenological and psychophysical evidence suggest the following tripartite model:

(I) encoding of unscaled (relative) depth that underlies the perception of object shape and layout;
(II) encoding of exocentric distances (scaled depth) optimized for near viewing only (less than 2 m);
(III) encoding of egocentric distance only, optimized for ambulatory distances (action space and beyond; greater than 2 m) without encoding of exocentric distances (scaled depth) or unscaled depth (shape and layout).

In the next three subsections, I will outline the implications of this model in terms of adaptive significance, neurophysiology and phenomenology.

### (d) Tripartite encoding of 3D space: adaptive, psychophysical and evolutionary significance

From an evolutionary lens, a full, scaled, master representation of 3D objects and space is unnecessary for many visually guided behaviours. There would certainly have been no selective pressure to evolve such a unified representation early in the evolution of visual function (figure 3). Instead, the selective pressure would have been to develop encodings adapted to the suite of visuo-motor capacities available to the animal. For example, the awareness of a space partitioned into regions (or rudimentary objects) ordered in depth (figure 4*a*), with the capacity to sense the distance to one or more of these regions/objects could support basic real-time planning of visually guided locomotion and navigation, even without awareness of 3D object shape or layout. Only animals with more advanced visuo-motor and cognitive apparatus that can support more complex real-time behaviour and planning (e.g. identification, recognition and visual orientation) would benefit from an awareness of 3D object shape and spatial layout. Similarly, only organisms with the motor apparatus for fine-grained visually guided manual behaviours (grasping, object manipulation and organization) would benefit from encodings of exocentric distances (scaled depth) that can support grasping, manipulation, etc. Moreover, scaled-depth encodings would only be adaptively significant in the personal (reach) space of the agent where fine-grained manual interaction occurs. They are not critical in action space and beyond, where encodings that simply provide information regarding the egocentric distance to an object or location of interest will suffice to support planning of ballistic (e.g. throwing, lunging) or locomotor/ambulatory behaviours (e.g. approach, retreat, navigation).

The consideration of the psychophysical operationalization of visual depth cues also supports the logic of a tripartite dissociation optimized for different regions of space. Derivation of scaled depth (exocentric distances) is thought to primarily rely on the scaling of binocular disparity by binocular distance cues. There has long been debate on the effectiveness of binocular distance cues (e.g. vergence, vertical disparity) for derivation of egocentric distance (see [82,83]). Even if these cues were valid, disparity scaling by vergence (or vertical disparity) would likely only be effective within reach space (1–2 m; [38,83]). This implies that available visual information restricts effective derivation of scaled intra- and inter-object distances to a limited region of space near the observer. By contrast, tasks requiring judgements of egocentric distance to targets in action space and beyond (greater than 2 m; e.g. blind walking to previewed targets) are thought to rely on ground plane information, perspective scaling and declination from eye level (see [48,68,84,85]), which are mostly useful only for distances outside the personal space of the observer (greater than 2 m).

### (e) Tripartite encoding of 3D space: neurophysiology

The hypothesis that the perception of 3D space in humans is based on a tripartite encoding is also supported by neurophysiological evidence. The well-established division of the primate visual pathways into the ventral (temporal) and dorsal (parietal) streams of processing [86,87], and the locus of areas underlying navigation and locomotion in the allocortex (entorhinal cortex and parahippocampal regions), is supportive of the view that distinct neural substrates underlie encodings of 3D shape and layout, exocentric distance (in reach space) and egocentric distance (at a locomotor or navigational scale).

The dorsal stream, particularly posterior parietal areas, is well established as the locus of transformation of visual information into a format that guides manual action, and therefore the posterior parietal areas are likely the substrates underlying the encoding of scaled depth (exocentric distances) within near space. Based on the tripartite encoding hypothesis, this suggests that the perceptual phenomenology associated with the impression of stereopsis and realness (object solidity, tangibility, negative spatial separation) originates in the parietal cortex. Consistent with this prediction, recent neuroimaging evidence [88,89] reveals selective activation of dorsal visual areas (posterior parietal cortex) for contrasts between conditions where the impression of stereopsis is present (stereoscopic images, monocular aperture viewing of single pictures) and conditions where it is absent (binocular viewing of single pictures). The fact that the same parietal regions are activated under both binocular and monocular stereopsis provides neurophysiological support to the view that the phenomenology of object solidity, tangibility and spatial separation that underlies our impression of realness of a 3D scene derives from cortical mechanisms that are independent of the specific cues (e.g. binocular disparity) that activate them. The fact that this region of the brain also underlies visuo-motor control of reaching and grasping lends credence to the link between the phenomenology of stereopsis and realness and the awareness of the capacity for motor interaction [13].

Existing neurophysiological evidence that has examined the neural correlates of the perception of 3D shape from various cues (disparity, texture, shading, etc.) suggests that the potential locus for the encoding of 3D object shape is in dorsal aspects of the extrastriate cortex (V3a) as well as early aspects of the temporal cortex, particularly the occipito-temporal cortex and areas extending to posterior temporal regions [90–94]. The area most typically highlighted for 3D shape recognition is the lateral occipital cortex rather than areas such as the posterior parietal cortex associated with guidance of manual action (though see [92,95]). Area V3a, which projects to both ventral and dorsal streams, is a potential junction where these two types of 3D encodings might start to diverge. No studies have been conducted to specifically investigate the potential difference in neural substrates between the perception of unscaled 3D structure (shape and layout) versus scaled 3D structure (exocentric distances). Testing stimuli where 3D structure is specified by binocular disparity cannot distinguish between scaled and unscaled 3D perception (see [88,89]) and doing so with other stimuli remains a challenge.

The main substrates encoding space for spatial navigation and planning at the ambulatory scale are thought to be the medial aspects of the inferior temporal cortex (entorhinal cortex and parahippocampal areas), as identified in both rodents and humans. In rodents, spatial encoding for navigation is thought to be based on the grid cells of the medial entorhinal cortex, though their specific role is still a matter of debate (see, for example, [96]). Grid-cell-like encodings have also been identified in human entorhinal cortex when subjects are engaging in navigation and locomotor tasks [97]. Also, the adjacent parahippocampal areas of the temporal cortex have been associated with encoding of spatial layout in humans in fMRI studies, with highest activations for images of outdoor scenes, but not 3D objects alone or relative layout of objects without spatial context [98], and these areas are not implicated in object recognition or memory [99]. Aspects of the posterior parietal cortex are also implicated in navigation (e.g. [100,101]), but on the basis of efferent inputs from medial temporal areas such as entorhinal cortex, rather than directly from visual cortex, suggesting that these areas are involved in converting spatial coding instantiated in the entorhinal cortex into action-relevant encodings [101].

Importantly, in contrast to neural substrates for 3D shape recognition and visual guidance of manual action, which are located in neocortex (temporal and parietal), the substrates that underlie visually guided locomotion are part of allocortex, which is an evolutionarily earlier division of the brain. This further supports the thesis that spatial encodings for perception of egocentric distance, which is crucial for real-time visual guidance of locomotion, are distinct from those underlying perception of 3D shape as well as those underlying the perception of exocentric distance (scaled depth in personal space), and that the perception of egocentric distance is likely the earliest of the 3D spatial competencies to evolve.

### (f) Tripartite encoding of 3D space: phenomenology

Psychophysical investigations always involve descriptions of perceptual 3D space as consisting of entities (surfaces, solid objects) and spatial attributes (direction, distance, depth, location) described in terms of Euclidean geometry in a Cartesian or spherical coordinate frame. This view leads to an assumption that the perceptual system in effect delivers a depth or range map of spatial coordinates (akin to the outputs of SLAM or light detection and ranging (LIDAR)) or perhaps a constructive solid geometry (CGS) model of it. While operationalizing perceptual space in this manner is no doubt critical for conducting psychophysical investigations, it is important to understand that ascribing these geometric entities and attributes to perceptual space and objects is simply that—an operationalization—and not to be reified as the actual constituents of the information content of the spatial encodings that determine how we phenomenologically experience 3D space.

Phenomenological analysis suggests that the perceptual entity that we psychophysically operationalize as a ‘surface’, and the perceptual attribute that we operationalize as ‘distance’, are not constructs that can be defined simply by geometry. Rather, in perception, these entities and attributes possess agent-centric content. They have a constitutively embedded content of behavioural anticipation and agency. We do not perceive the distance to an object as a quantitative geometric value, but as an anticipatory attribute rooted in motor agency. The content of ‘perceptual distance’ is therefore far more complex than just geometric distance, even though in psychophysical operationalization we can usefully reduce the former to the later.

The more complex perceptual content of the spatial attribute ‘distance’ is highlighted by the classic blind-walking paradigm. In blind walking, the observer previews an object and then walks blindfolded for a distance matching the perceptually judged distance, which observers can do accurately for at least 25 m [39]. In doing the task, it is not as though one makes a mental note of a quantitative estimate from visual perception, and then applies this to derive the number of steps or duration required to blind-walk a matched distance. Instead, the subjective phenomenology of the task is that one has an embodied anticipatory encoding of the distance to the object, where, during the blind-walking phase, there is a sort of ‘embodied cancellation’ of this anticipated distance based on an idiothetic record of distance traversed. In this way, the awareness of distance to an object is similar to how we have proprioceptive and anticipatory awareness of the location of our hand with respect to the location of our nose, which we can touch with eyes closed. From this perspective, it would make sense that the awareness of egocentric distance originates in the idiothetic encodings identified in the areas of allocortex related to locomotion and navigation, for example, grid cells, rather than directly (via inference) from visual input alone. The nature of grid-cell firing logic, in that grid cells ‘mark’ traversed distances and that the spacing of the marked positions (nodes) appear to be dependent on sensory input [96] is suggestive of an early substrate for the anticipatory encoding of egocentric distance, rather than simply a substrate for path integration. This view is also echoed in Warren [48, p. 169]: ‘the visually perceived distance to a thing derives from the proprioception entailed in walking to it, that is, from the distance sensed by the human odometer’.

This idea of the perception of spatial attributes as intrinsically anticipatory and embodied is also echoed in the phenomenology of stereopsis which gives rise to the explicit feeling of agency towards objects (tangibility), the impression of a palpable separation between things (negative space) and an overall realness. This feeling is absent when viewing pictures normally and coincides with the fact that pictorial space lacks an optically specified scale (figure 4), which is critical for motor interaction.

Similarly, the perception of 3D surface shape should not be considered to be simply the awareness of the locus of points (SLAM-like) or a compact geometric description of such points (e.g. polygonal mesh or NURBS^8^). Rather, a perceptual surface is a complex information structure that provides an intrinsic anticipation of how the entity will interact with tactile exploration or manipulation, and furthermore is likely to have embedded more complex perceptual content such as nested shape histories (see [102]).

The view espoused above is perhaps most strongly echoed in the Gibsonian notion of affordances, which evolved from earlier concepts from the *Gestalt* tradition and ethology, for example,  the notion of *Aufforderungscharakter* (demand character) [103]. However, the nature of Gibson's affordances and how they link to what is perceived as objects, surfaces, space and layout have been a source of debate and controversy (see, for example, [48,104]). Gibson's own writings seem to emphasize the use of the term affordances to refer to higher-order perceptual constructs, such as a surface appearing walkable or climb-up-able, openings appearing passable, or objects appearing wieldable, throwable, or graspable. As Warren [48] points out, it is unclear what Gibson intended to be consciously presented in spatial perception, so there are three possibilities regarding what is peceived: (1) only higher-order affordances, and not spatial attributes; (2) only spatial attributes (distance, slant, depth, curvature, etc.), from which affordances are inferred indirectly; or (3) both affordances and spatial attributes are directly and consciously perceived. There is some indication that Gibson held the view that only affordances are consciously perceived while perceptual attributes such as layout and distance are only implicitly coded: ‘What animals need to perceive is not the layout as such but the affordances of the layout’ [9, p. 150]. Furthermore, Gibson argued that affordances were ‘objective’ and ‘real’ and not related to ‘subjective’ phenomenology [9], which contrasts with the view proposed here, where the term phenomenology is used to encompass all that is perceived. But there is ambiguity here as well, because Gibson specifically also  stated that 'an affordance is neither an objective property nor a subjective property' [9, p. 129], which is consistent with the idea that spatial encodings that we phenomenologically experience have content that can only be defined relationally between the external reality and the sensorimotor competencies of the agent [47,48].

The proposal offered here differs from the Gibsonian idea that only affordances are perceived and that the anticipatory content of affordance is available only at the macro level of behaviour (walkable surfaces, sittable objects, throwable objects). Instead, it claims that the perception of both spatial attributes and higher-order affordances is consciously perceived and that these form part of our perceptual phenomenology. Affordances, as described in Gibson [9], can then be seen as essentially higher-order emergent perceptual attributes deriving from the fact that the fundamental spatial entities and attributes in perception (surfaces, distance, size, shape and layout) are themselves encoded in terms of proprioceptive/idiothetic variables, where the latter underlie the specific sorts of phenomenology that we experience for various perceptual entities and attributes (e.g. where objects seem tangible and depth separations appear ‘real’ (stereopsis), where distance provides an anticipatory impression of required locomotion, or where a surface anticipates the tactile sensation). In other words, the sensorimotor anticipatory aspect is constituted within the *microstructure* of perceived spatial entities and variables.

Uncovering and modelling the embodied and anticipatory structure of the basic attributes of space will no doubt require a major programme of research. However, the considerations so far, at the very least, help sketch out broadly what distinct spatial encodings can be identified in human observers based on evolutionary, phenomenological and psychophysical considerations.

## 5. Conclusion

The most popular model of 3D perception of the last 25 years (summarized in [5]) has largely avoided consideration of a range of important phenomenological and psychophysical observations that fundamentally challenge its underlying assumptions. Moving forward toward a more comprehensive understanding of 3D perception will require jettisoning cherished assumptions: (1) that 3D perception entails an ‘ideal-observer’ inference of an objective 3D representation, (2) that this representation is unitary and veridical, and (3) that spatial encodings can simply be modelled on our understanding of the geometry of the external world, without consideration of how the agents' own sensory and motor capacities are constitutively embedded in that structure in such a way that provides an anticipatory encoding of space.

Also important is to acknowledge that the evolution of visuo-spatial encodings was necessarily driven by adaptive pressures that do not lead to the need for the inference of the 'objective' external world. Careful consideration is required in understanding what exactly it is that we are modelling (mental content versus mind-independent structure) and how we should approach such modelling (phenomenology versus psychophysics). Importantly, we must not confuse psychophysical operationalizations of entities or attributes of 3D space with the underlying encodings that achieve the anticipatory structure we perceive in 3D perception.

## Data Availability

This article does not contain any additional data.

## Endnotes

1 Note that while the more recent work of Koenderink and collaborators embraces the phenomenological approach, the interpretations that they have published relevant to the discussion here [12,26] relied on concepts from inferentialist cue-integration models.

2 Synoptic viewing involves binocular viewing of a single pictorial image through a synopter. A synopter uses lenses and/or prisms to deliver identical images to both eyes, such that no retinal disparities are present for the viewed object or scene (see [12,31]).

3 Monocular aperture viewing involves viewing a single picture with one eye though a small reduction aperture such that the edges of the picture are obscured (see [32]).

4 Koenderink and collaborators [12] have provided data from two obervers (the authors) showing that perceived depth relief of objects is shallower under binocular compared with synoptic viewing of pictorial images. However, our own recent tests of naive observers using similar methods (gauge figure task) reveals no difference in perceived magnitude of depth between monocular aperture and binocular viewing.

5 But not the sensorimotor account of O'Regan & Noë [52] where perception is viewed as a set of sensory-motor contingencies operating an objective external structure, where the organism ‘acts out' its perceptions (see [53] for a critique).

6 For readers unfamiliar with the details of the historical developments in colour perception, an excellent lay introduction can be found in chapter 8 of Hubel's introductory text on vision [55].

7 Simultaneous localization and mapping (SLAM). A method in computer vision where coordinates of points making up objects and surfaces in a scene are derived from multiple 2D pictorial images of the scene (see the Introduction to this issue) [62].

8 NURBS, non-uniform rational basis splines.
